# Naphtalimide-Based Bipolar Derivatives Enabling High-Efficiency OLEDs

**DOI:** 10.3390/molecules28166027

**Published:** 2023-08-12

**Authors:** Raminta Beresneviciute, Prakalp Gautam, Mangey Ram Nagar, Gintare Krucaite, Daiva Tavgeniene, Jwo-Huei Jou, Saulius Grigalevicius

**Affiliations:** 1 Department of Polymer Chemistry and Technology, Kaunas University of Technology, Radvilenu Plentas 19, LT50254 Kaunas, Lithuania; 2 Department of Materials Science and Engineering, National Tsing Hua University, No. 101, Section 2, Guangfu Rd., East District, Hsinchu 30013, Taiwan

**Keywords:** bipolar derivatives, thermal stability, green emission, glass transition temperature, organic light-emitting diode, high efficiency

## Abstract

Organic light-emitting diodes (OLEDs) have revolutionized the world of technology, making significant contributions to enhancing our everyday lives. With their exceptional display and lighting capabilities, OLEDs have become indispensable in various industries such as smartphones, tablets, televisions, and automotives. They have emerged as a dominant technology, inspiring continuous advancements, and improvements. Taking inspiration from the remarkable advancements in OLED advancements, we have successfully developed naphtalimide-based compounds, namely RB-08, RB-09, RB-10, and RB-11. These compounds exhibit desirable characteristics such as a wide bandgap, high decomposition temperatures (306–366 °C), and very high glass transition temperatures (133–179 °C). Leveraging these exceptional properties, we have harnessed these compounds as green emitters in the aforementioned devices. Among the various fabricated OLEDs, the one incorporating the RB-11 emitter has exhibited superior performance. This specific configuration achieved maximum power efficacy of 7.7 lm/W, current efficacy of 7.9 cd/A, and external quantum efficiency of 3.3%. These results highlight the outstanding capabilities of our synthesized emitter and its potential for further advancements in the field.

## 1. Introduction

Organic light-emitting diodes (OLEDs) transcend the capabilities of conventional diodes, excelling in performance, durability, and manufacturing processes [1,2,3,4]. In addition to these achievements, OLEDs possess a plethora of remarkable features that set them apart from other diode types [5,6]. Notably, OLEDs offer self-illumination, wide viewing angles, rapid response times, high color contrast, low operating temperatures, exceptional color rendering indexes (CRIs), soft and diffused emission, full-spectrum color reproduction, color tunability, planar design, spectrum tailoring, unbreakable construction, lightweight and thin form factor, flexibility, transparency, ease of molecular design, the utilization of sustainable materials, energy-saving characteristics, human- and eco-friendliness, and low driving voltages [7,8,9]. These distinctive attributes establish OLEDs as a disruptive technology. As a result, it is anticipated that OLEDs will eventually supplant other diode types, particularly light-emitting diodes (LEDs), in the near future [10,11,12].

For this reason, organic electroactive materials are extensively synthesized and studied as components of the aforementioned devices. Bipolar organic derivatives can be used as materials of emitting layers of OLEDs. Some naphtalimide-based derivatives as electroactive materials are already described in the scientific literature [13,14,15]. In this study, we present new potential emitters containing naphtalimide cores as electron acceptors and carbazole or arylcarbazole fragments as electron donors. Some of the new materials demonstrated promising electroluminescent characteristics as emitters in the OLED devices.

## 2. Results and Discussion

**Figure 1:**
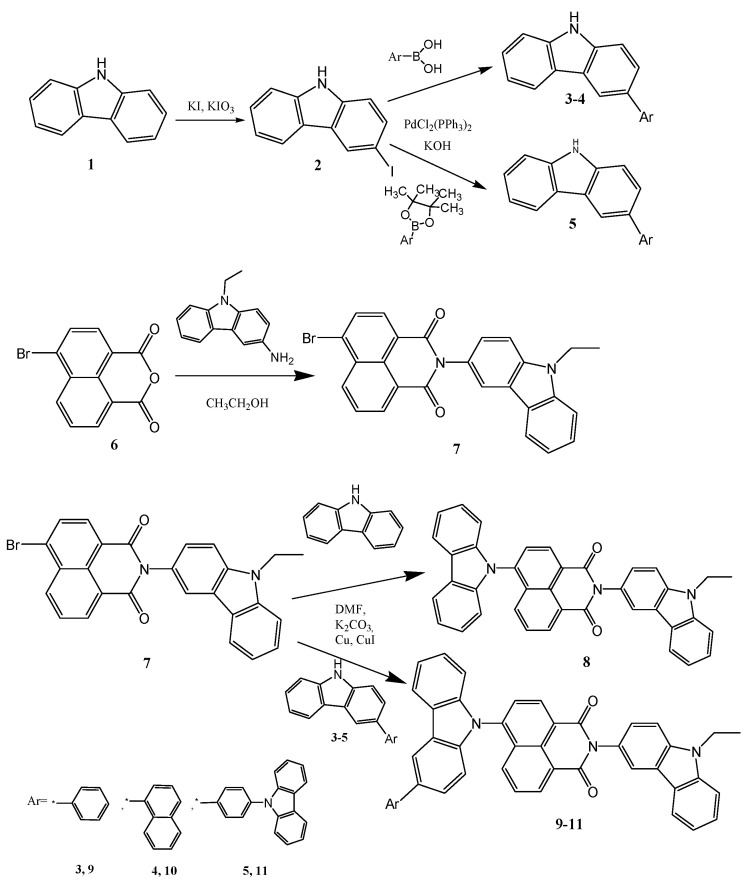
Synthetic pathways of the compounds RB-08 (**8**), RB-09 (**9**), RB-10 (**10**), and RB-11 (**11**).

The synthesis of naphtalimide-based compounds was accomplished by the synthetic approach depicted in Figure 1. 3-Iodo-9H-carbazole (**2**) was obtained from 9H-carbazole via a procedure by Tucker. Then, intermediate carbazole derivatives (**3**–**5**) were prepared via the Suzuki reaction of the 3-iodo-9H-carbazole (**2**) with an excess of equivalent boronic acid or boronic acid pinacol ester in a basic environment, with a palladium catalyst. Derivative **7** was synthesized as a key compound from commercially available materials 4-bromo-1,8-napthalic anhydride (**6**) and 3-amino-9-ethylcarbazole. The objective compounds **8**–**11** were synthesized via Ullmann coupling the crucial material’s reaction **7** with 9H-carbazole or the intermediate carbazole-based derivatives **3**–**5**, correspondingly. The prior reactions occurred in dimethylformamide (DMF) using 18-crown-6 as a catalyst. Novel synthesized derivatives have been discovered using mass spectrometry and NMR spectroscopy. The data were found to be consistent with the postulated structures. At room temperature, the derivatives were soluble in common organic solvents such as chloroform, DMF, or THF.

### 2.1. Characteristics of the Compounds

#### 2.1.1. Photophysical Properties

**Figure 2:**
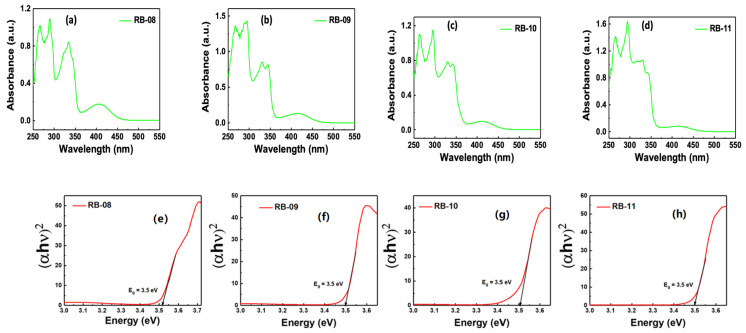
The ultraviolet–visible absorbance (UV abs) spectra for compounds RB-08, RB-09, RB-10, and RB-11 (**a**–**d**). The corresponding Tauc plot illustrates the absorption wavelength and bandgap (**e**–**h**).

Under ambient conditions, the ultraviolet absorbance (UV abs) spectra of the compounds RB-08, RB-09, RB-10, and RB-11 were examined using tetrahydrofuran (THF) solvent. Figure 2a–d show the absorbance spectra of the prepared solutions measured using a quartz cuvette. The UV spectra of the objective materials can be found to be extremely similar. Only the intensity of the peak at about 410 nm depends on the substituent at the fourth position of the naphtalimide core. Furthermore, a Tauc plot was constructed (Figure 2e–h) using the absorption wavelength and intensity data. The Tauc plot was generated by employing the following Equation (1) [16]:

(αhν)^2^ = A(hν − E_g_) (1)

where hν is the photon energy (1240/wavelength), and α is the absorption coefficient, A is a constant, and E_g_ is the optical bandgap.

It could be observed from the results that all the objective materials have very similar optical bandgaps (E_g_) of about 3.5 eV.

**Figure 3:**
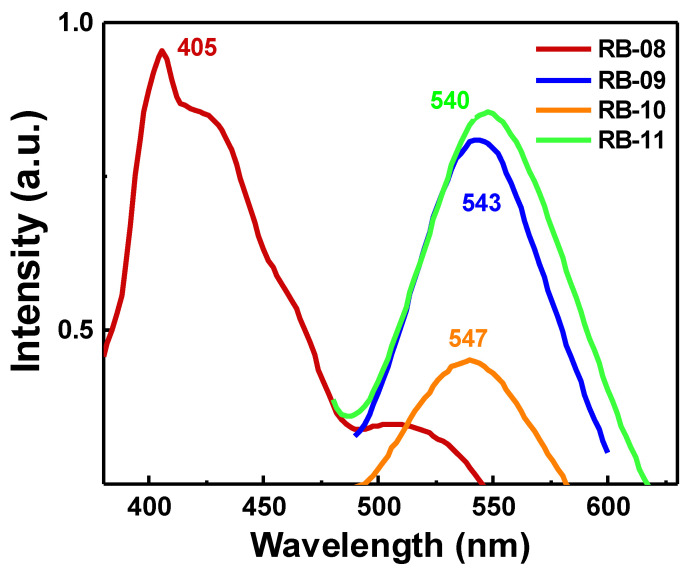
Photoluminescence (PL) spectra of compounds RB-08, RB-09, RB-10 and RB-11.

The photoluminescence (PL) spectra of compounds RB-08, RB-09, RB-10, and RB-11 are depicted in Figure 3, illustrating the peak emission wavelengths within the range of 400–600 nm. The emission maxima of the derivatives also depend on the substituent at the fourth position of the naphtalimide core. Carbazol-9-yl-substituted derivative RB-08 has a maximum emission level at 405 nm. Other compounds, having 3-arylcarbazol-9-yl substituents, demonstrate similar maxima of green emissions in the range of 540–547 nm due to bigger conjugation levels in the molecules as compared with RB-08.

#### 2.1.2. Electrochemical Properties

**Figure 4:**
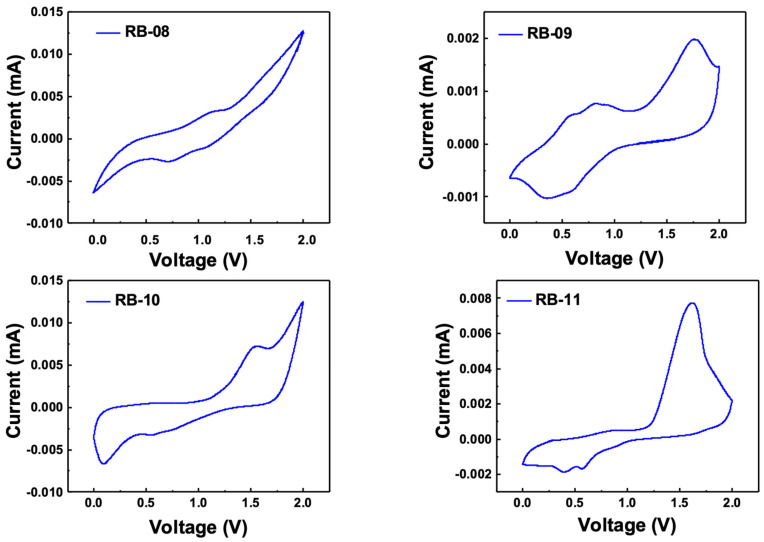
Cyclic voltammetry scans for calculation of HOMO levels of compounds RB-08, RB-09, RB-10, and RB-11.

Cyclic voltammetry (CV) measurements were conducted to estimate the electrochemical characteristics of compounds RB-08, RB-09, RB-10, and RB-11. The results of these measurements are presented in Figure 4.

The calculation of the HOMO levels was performed using Equation (2) [17]:

E_HOMO_ = −[4.4 + *E^ox^_onset_*] (2)

where *E^ox^_onset_* is the oxidation onset potential of a material, and 4.4 eV is the Fc^+^/Fc potential.

The calculation of the LUMO levels was performed using Equation (3):

E_LUMO_ = E_HOMO_ + E_g_ (3)

The bandgap (E_g_) was calculated using the Tauc plot, and the values obtained were utilized to determine the energy levels of the lowest unoccupied molecular orbital (HOMO). For RB-08, RB-09, RB-10, and RB-11, the measured HOMO energy levels were determined to be −5.7, −5.6, −6.1, and −5.6 eV, respectively. Similarly, the LUMO levels were identified to be −2.2, −2.1, −2.6, and −2.1 eV for RB-08, RB-09, RB-10, and RB-11, respectively. These results indicate that the HOMO and LUMO levels of the derivatives are suitable for green emitters.

#### 2.1.3. Thermal Properties

**Figure 5:**
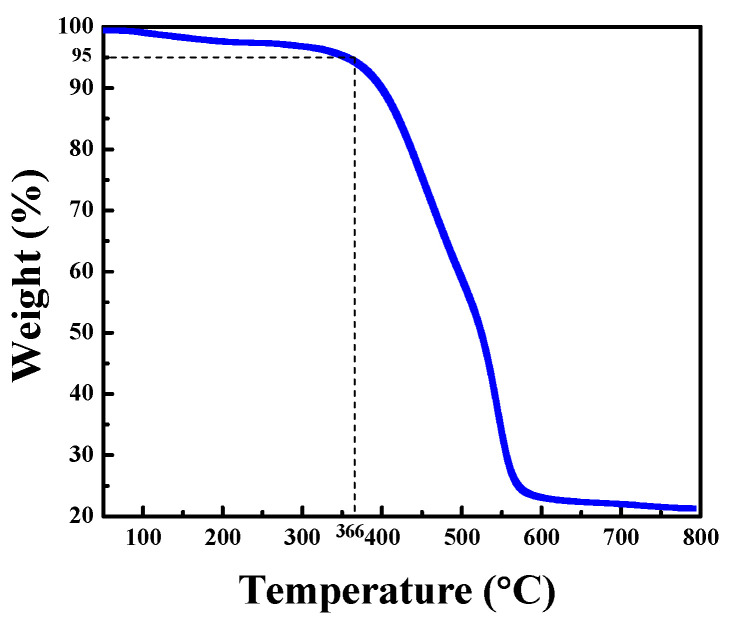
TGA curve of compound RB-11 at the heating rate: 10 °C/min.

The heating behavior of the synthesized compounds RB-08-11 was investigated using DSC and TGA in a nitrogen atmosphere. It was discovered that all of the target compounds have extremely good thermal stability. The 5% weight loss temperatures (T_d_) for derivatives RB-08, RB-09, and RB-10 were 306, 355 and 373 °C, respectively, as confirmed via TGA with a heating rate of 10 °C/min. Compound RB-11 was also highly stable under heating with a T_d_ of 366 °C, as it is demonstrated with its TGA curve as an example in Figure 5.

**Figure 6:**
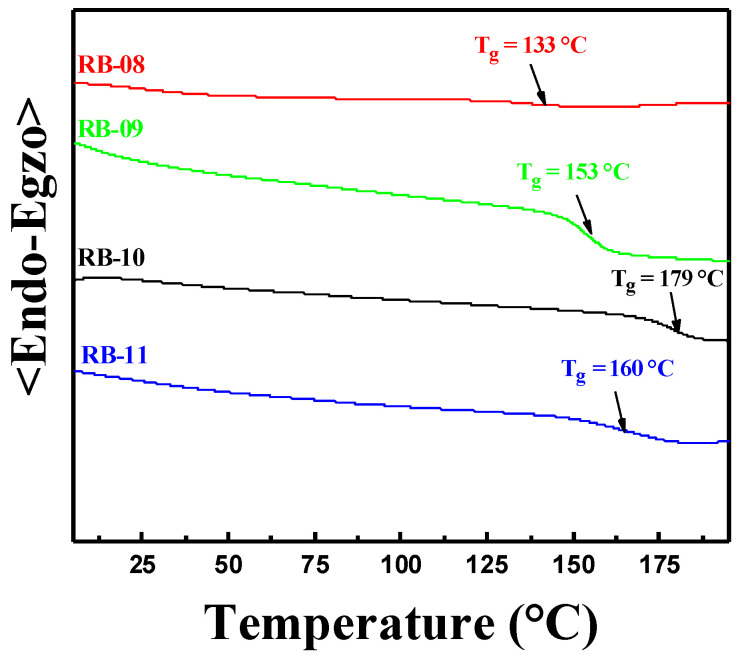
DSC second heating curves of compounds RB-08, RB-09, RB-10, and RB-11.

Figure 6 depicts the DSC thermograms of the objective compounds’ second heating. After synthesis, all of the derivatives were produced as amorphous compounds with extremely high glass transition temperatures (T_g_). Compound RB-10 demonstrated the highest glass transition temperature of 179 °C. The other derivatives, RB-08, RB-09 and RB-11, showed only slightly lower glass transition temperatures of, respectively, 133, 153, and 160 °C. Overall, the TGA and DSC results show that the materials are suitable for use in electroactive layers of OLED devices.

### 2.2. Structure and Characterization of Electroluminescent OLED Devices

**Figure 7:**
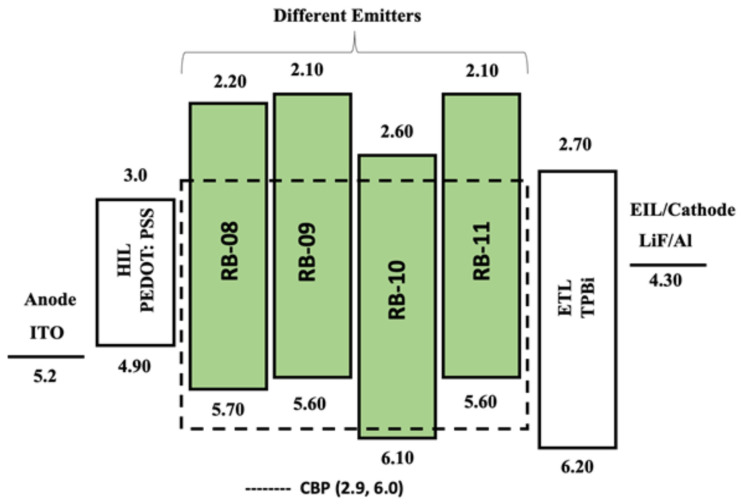
The energy-level diagram of the solution-processed green OLED devices containing emitters RB-08, RB-09, RB-10, and RB-11 doped in the CBP host matrix. Dashed lines show HOMO-LUMO levels of the CBP host.

We conducted a study to explore the potential of RB-08, RB-09, RB-10, and RB-11 compounds in optoelectronic devices, inspired by their exceptional emissive behavior, optical properties, and high solubility in solvents. Our investigation involved creating a series of multi-layered solution-processed OLED devices. We used different doping concentrations and host-based strategies to evaluate the electroluminescent (EL) characteristics of these compounds. To achieve optimal EL performance, we employed a specific device configuration. The configuration consisted of the following layers: ITO (150 nm)/PEDOT:PSS (40 nm)/CBP:x wt% RB-08, RB-09, RB-10, or RB-11 (x = 5.0, 7.5, 10, and 100) (20 nm)/TPBi (35 nm)/LiF (1 nm)/Al (200 nm). This configuration was chosen to facilitate efficient charge-carrier transport and exciton generation, as illustrated in Figure 7. The anode material was ITO, while Al served as the cathode material. PEDOT:PSS was used as the hole-transporting material, CBP as the host material, TPBi as the electron-transporting material, and LiF as the electron-injection material. By employing this device configuration and investigating various doping concentrations and host-based strategies, we aimed to assess the EL characteristics of RB-08, RB-09, RB-10, and RB-11 compounds in OLED devices.

The device without doping exhibited poorer electroluminescent properties and brightness, likely due to an imbalance in the injection and transportation of charge carriers in the emissive layer [18,19]. It is crucial to achieve a balanced distribution of charge carriers in order to improve the performance of OLEDs, and an efficient host–guest energy transfer system can assist in achieving this. In this study, OLED devices were created by incorporating different concentrations of emitters into the host matrix. The optimization of emitter concentration plays a vital role in enhancing the efficiency, stability, and lifespan of OLED devices. The selection of a CBP host was based on several considerations. Firstly, its HOMO-LUMO energy level is compatible with those of the emitting molecules. Secondly, its bipolar nature aids in balancing the charge carriers within the emissive layer. Lastly, its high triplet energy helps prevent back-energy transfer from the dopant [20].

The electroluminescence (EL) characteristics of the devices using the emitters RB-08, RB-09, RB-10, and RB-11 are depicted in Figures S1–S4, along with the corresponding characteristics of the devices. Each figure consists of the following components: (a) EL spectra, (b) current density–voltage characteristics, (c) luminance–voltage characteristics, (d) power efficacy–luminance characteristics, (e) current efficacy–luminance characteristics, and (f) luminance–current density characteristics. Furthermore, Table S1 provides a summarized overview of the device characteristics.

**Figure 8:**
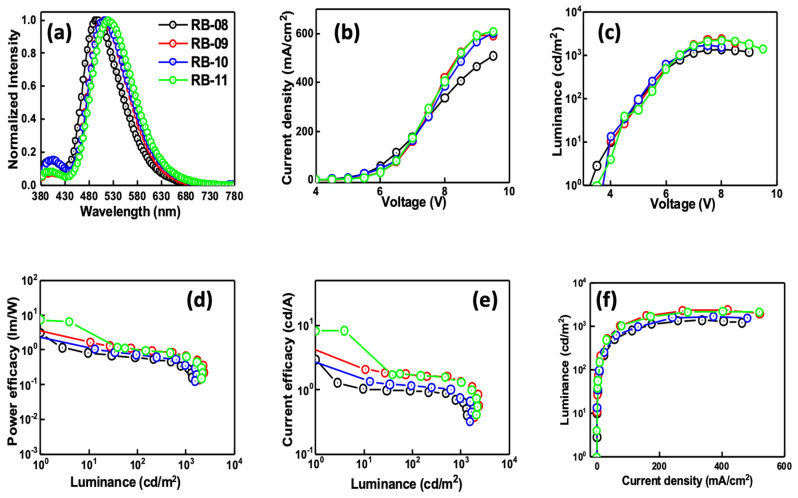
(**a**) EL spectra, (**b**) current density–voltage, (**c**) luminance–voltage, (**d**) power efficacy–luminance, (**e**) current efficacy–luminance, and (**f**) luminance–current density characteristics of the devices using RB-08, RB-09, RB-10, or RB-11 as emitters at 5.0 wt% doping concentration in CBP host matrix.

The host material played a crucial role, and among all the devices and emitters, the one with the 5 wt% RB-11 emitter demonstrated superior performance. This device exhibited maximum power efficacy (PE_max_) of 7.7 lm/W and maximum current efficacy (CE_max_) of 7.9 cd/A with a driving voltage of 4.1 eV. Additionally, the device based on RB-11 showed the highest EQE_max_ of 3.3%. The detailed EL characteristics of the best devices based on RB-08, RB-09, RB-10, and RB-11 are summarized in Table 1 and Figure 8.

The EL spectra exhibit peaks in the range of 480–550 nm, indicating green emissions. When a single peak is observed, it indicates that there is a full transfer of energy between the host and guest materials. In many instances, the emission wavelength in EL matches the PL spectra of the corresponding emitters. This alignment signifies that the emission originates from the material itself. The device utilizing emitter RB-11 achieved Commission Internationale de L’Eclairage (CIE) coordinates of (0.29, 0.52). This successful outcome indicates the creation of an efficient and stable green OLED device, with a maximum EL peak at 520 nm. An optimal balance between efficient energy transfer and charge transport results in improved device performance [21].

## 3. Experimental Section

### 3.1. Instrumentation

An HP-8453 diode array spectrometer (Agilent Technology Inc., Hachioji, Tokyo, Japan) was used to measure the UV-vis absorption spectra of the compounds. Furthermore, the Tauc plot was obtained using the absorption wavelength. An Aminco-Bowman series 2 luminescence spectrometer (Agilent Technology Inc., Hachioji, Tokyo, Japan) was used to record the photoluminescence (PL) spectra. To determine the highest occupied molecular orbital (HOMO) levels, cyclic voltammetry (CV) was performed using the CH1604A potentiostat (Annatech Co., Ltd., Taipei, Taiwan). Thermo-gravimetric analysis (TGA) was performed on a TGAQ50 apparatus (Verder Scientific Haan, Haan, Germany). Differential scanning calorimetry (DSC) measurements were carried out using a Bruker Reflex II thermos-system (Bruker, Berlin, Germany). The TGA and DSC curves were measured in a nitrogen atmosphere at a 10 °C/min heating rate.

### 3.2. Device Fabrication

The OLED devices were fabricated using a pre-sputtered ITO glass substrate. The substrate was cleaned for 30 min each with acetone and isopropyl alcohol (IPA) at temperatures of 50 and 60 °C. Consequently, the substrates were moved to a preheated UV chamber and exposed to UV light for 10 min. The layer deposition took place in a glove box with an inert atmosphere. The hole injection layer (PEDOT:PSS) was spin-coated for 20 s at 4000 rpm before heating the substrates for 10 min at 130 °C. The cooled substrates were then spin-coated with an emissive layer for 20 s at 2500 rpm. The next steps involved the thermal evaporation of the electron-injecting/transporting layer and aluminum cathode in a vacuum of 10-6 torr. The substrates were kept under a vacuum in a mini chamber within the glove box to prevent them from deteriorating until they were ready to be tested individually. All testing procedures were performed in a completely dark room under ambient conditions. For recording the current density–voltage–luminance (J-V-L) characteristics, the CS-100A luminescence spectrophotometer was employed. Meanwhile, the power efficacy–luminance–current characteristics were recorded using the PR-655 spectrophotometer. The current–voltage (I-V) characteristics were measured using a Keithley voltmeter. The device area was determined to be 0.09 cm^−2^. To calculate the EQE of the devices, the method outlined in the relevant literature was carefully followed throughout the testing and analysis process [22].

### 3.3. Synthetic Procedures of the Materials

The 3-iodo-9H-carbazole (**2**) was prepared from commercially obtained 9H-carbazole by using methodology of the Tucker iodination reaction.

3-Phenyl-9H-carbazole (**3**). Phenylboronic acid (0.52 g, 4.27 mmol), 3-iodo-9H-carbazole (1.00 g, 3.41 mmol), potassium hydroxide (0.95 g, 16.96 mmol), and PdCl_2_(PPh_3_)_2_ (0.09 g (0.13 mmol) were added into mixture of THF (10 mL) and degassed water (1 mL). The formed reaction mixture was stirred and refluxed for 3 h under nitrogen. The progress of the reaction was checked via TLC control. After the reaction, product was extracted using chloroform, which was then dried with anhydrous Na_2_SO_4_. The crude product was adsorbed into silica gel and purified via silica gel column chromatography. A mixture of ethyl acetate/hexane (1:5 by vol.) was used as an eluent. The yield of the 3-phenyl-9H-carbazole (**3**) was 0.85 g, which was obtained as a white material.

3-(1-Naphtyl)-9H-carbazole (**4**). Here, 1 g (3.41 mmol) of 3-iodo-9H-carbazole, naphthalene-1-boronic acid (0.73 g, 4.23 mmol), PdCl_2_(PPh_3_)_2_ (0.09 g, 0.13 mmol) and 0.95 g (16.96 mmol) of KOH were mixed with 10 mL of THF and degassed water (1 mL). The reaction mixture was stirred and refluxed for 3 h under nitrogen. After TLC control, the reaction was finished. Organic products were extracted using chloroform, which was then dried over anhydrous sodium sulphate. The 3-(1-naphtyl)-9H-carbazole (**4**) was separated from the mixture via silica gel column chromatography. The ethyl acetate/hexane mixture (vol. ratio 1:5) was an eluent. The yield of the white material was 0.95 g.

3-[4-(Carbazol-9-yl)phenyl]-9H-carbazole (**5**). Here, 1 g (3.41 mmol) of 3-iodo-9H-carbazole, 0.09 g (0.13 mmol) of PdCl_2_(PPh_3_)_2_, 9H-carbazole-9-(4-phenyl) boronic acid pinacol ester (1.57 g, 4.3 mmol), and KOH (0.95 g, 16.96 mmol) were added into a mixture of THF (10 mL) and degassed water (1 mL).The mixture was refluxed under nitrogen for 3 h followed by TLC control. Organic products were extracted from the mixture using chloroform, which was then dried over anhydrous Na_2_SO_4_. The combined extract was dried over anhydrous Na_2_SO_4_. The material **5** was separated via silica gel chromatography using an ethyl acetate/hexane mixture (1:7 by vol.) as an eluent. The yield of the white material was 0.62 g.

N-(9-Ethylcarbazol-3-yl)-4-bromo-1,8-naphtalymide (**7**). Here, 1.58 g (7.50 mmol) of 3-amino-9-ethylcarbazole and 4-bromo-1,8-napthalic anhydride (1.90 g, 6.85 mmol) were added into 30 mL of ethanol. The mixture was refluxed for 24 h followed by TLC control. After the cooling of the reaction mixture, the product formed crystalline material, which was filtered and washed with hexane. The yield of the N-(9-ethylcarbazol-3-yl)-4-bromo-1,8-naphtalymide (**7**) was 2.76 g of brown powder.

N-(9-Ethylcarbazol-3-yl)-4-(carbazol-9-yl)-1,8-naphtalimide (**8**). Here, 0.50 g (1.07 mmol) of N-(9-ethylcarbazol-3-yl)-4-bromo-1,8-naphtalimide, 0.21 g (1.26 mmol) of 9H-carbazole, and 0.08 g (0.42 mmol) of 18-crown-6 were stirred in 10 mL of DMF at reflux under nitrogen. Then, potassium carbonate (0.58 g, 4.2 mmol), Cu (0.08 g, 1.26 mmol), and CuI (0.20 g, 1.05 mmol) were added stepwise. The mixture was left to react for 24 h. After TLC control, the inorganic materials were filtered off and the product was extracted using chloroform. The combined extract was dried over anhydrous Na_2_SO_4_. The crude product was purified via silica gel column chromatography using the mixture of ethyl acetate and hexane (vol. ratio 1:10) as an eluent. Yield: 0.082 g of yellow powder.

N-(9-Ethylcarbazol-3-yl)-4-[3-(1-naphthyl)carbazol-9-yl]-1,8-naphtalimide (**10**). Here, 0.50 g (1.07 mmol) of N-(9-ethylcarbazol-3-yl)-4-bromo-1,8-naphtalimide, 0.34 g (1.16 mmol) of 3-(1-naphtyl)-9H-carbazole, and 0.1 g (0.25 mmol) of 18-crown-6 were stirred in 10 mL of DMF at reflux under nitrogen. Then, potassium carbonate (0.58 g, 4.2 mmol), Cu (0.12 g, 1.89 mmol), and CuI (0.45 g, 2.36 mmol) were added stepwise. The mixture was left to react for 24 h. After TLC control, the inorganic materials were filtered off and the product was extracted using chloroform. The combined extract was dried over anhydrous Na_2_SO_4_. The crude product was purified via silica gel column chromatography using a mixture of ethylacetate and hexane (vol. ratio 1:15) as an eluent. Yield: 0.11 g of yellow powder.

N-(9-Ethylcarbazol-3-yl)-4-{3-[4-(carbazol-9-yl)phenyl]carbazol-9-yl}-1,8-naphtalimide (**11**). Here, 0.50 g (1.07 mmol) of N-(9-ethylcarbazol-3-yl)-4-bromo-1,8-naphtalimide, 0.58 g (1.39 mmol) of 3-[4-(carbazol-9-yl)phenyl]-9H-carbazole, and 0.05 g (0.20 mmol) of 18-crown-6 were stirred in 10 mL of DMF at reflux under nitrogen. Then, potassium carbonate (0.70 g, 5.10 mmol), Cu (0.13 g, 1.95 mmol), and CuI (0.48 g, 2.39 mmol) were added stepwise. The mixture was left to react for 24 h. After TLC control, the inorganic materials were filtered off and the product was extracted using chloroform. The combined extract was dried over anhydrous Na_2_SO_4_. The crude product was purified via silica gel column chromatography using a mixture of ethyl acetate and hexane (vol. ratio 1:7) as an eluent. Yield: 0.07 g of yellow powder.

## 4. Conclusions

A series of naphtalimide-based compounds (RB-08, RB-09, RB-10, and RB-11) were successfully synthesized and characterized. Their photophysical, electrochemical, and thermal properties were examined in regard to their prospective application in green OLED devices. UV-visible absorption spectra indicated strong absorption in the UV region, and Tauc plot analysis was utilized to determine the optical bandgap. Photoluminescence spectra exhibited peak emission wavelengths ranging from 400 to 550 nm, indicating green emission characteristics. Cyclic voltammetry measurements provided insights into the electrochemical properties of the compounds, and the calculated HOMO and LUMO energy levels confirmed their suitability as emitters with a 4,4’-bis(carbazol-9-yl)biphenyl (CBP) host. Furthermore, the materials were thermally and morphologically stable, with high breakdown temperatures and very high glass-transition temperatures. The thermal analysis demonstrated that the synthesized materials are well-suited for OLED applications. Furthermore, OLED devices were fabricated using the synthesized compounds as dopants in the CBP host matrix. The electroluminescence spectra of the devices displayed green emissions, and the devices exhibited favorable performance characteristics. Among the devices, the one incorporating a 5 wt% RB-11 emitter demonstrated superior performance, achieving maximum power efficacy of 7.7 lm/W, maximum current efficacy of 7.9 cd/A, and maximum external quantum efficiency of 3.3%. The CIE coordinates of (0.29, 0.52) of the RB-11-emitter-based device indicated an efficient and stable green OLED with peak emission at 520 nm. Finally, the synthesized naphtalimide-based compounds show promising potential as efficient green emitters for OLED applications. These cost-effective materials exhibit suitable photophysical, electrochemical, and thermal properties, making them suitable for a range of display and solid-state lighting applications. The further optimization and refinement of device parameters could potentially enhance performance and expand the applications of these materials in the field of organic optoelectronics.

## Figures and Tables

**Table 1:** Electroluminescence properties of RB-08-, RB-09-, RB-10-, and RB-11 (5.0 wt%)-based solution-processed OLED devices with CBP host matrix.

Emitter	Dopant Concentration (wt%)	Driving Voltage (V)	Operation Voltage (V)	Power Efficiency (lm/W)	Current Efficiency (cd/A)	EQE (%)	CIE	Max Luminance (cd/m^2^)
			@100, 1000 cd/m^2^ and Maximum					
RB-08	5	4.0	5.0/6.8/3.0	0.6/0.3/3.0	1.0/0.7/2.9	0.4/0.3/1.1	(0.22, 0.42)/(0.21, 0.36)/-	1359
RB-09	5	4.0	5.1/6.5/3.5	1.1/0.7/6.2	1.7/1.4/6.9	0.7/0.5/1.6	(0.27, 0.5)/(0.24, 0.45)/-	2364
RB-10	5	3.9	5.0/6.5/3.5	0.7/0.4/4.2	1.2/0.7/4.6	0.6/0.4/1.6	(0.26, 0.48)/(0.23, 0.41)/-	1687
RB-11	5	4.1	5.2/6.5/3.2	1.0/0.6/7.7	1.7/1.3/7.9	0.6/0.5/3.3	(0.29, 0.52)/(0.26, 0.46)/-	2212

## Data Availability

The data presented in this study are available on request from the corresponding authors.

## Supplementary Materials

The following supporting information can be downloaded at: https://www.mdpi.com/article/10.3390/molecules28166027/s1, Figure S1: The electroluminescent (EL) properties of the device with emitter RB-08 doped in CBP host matrix at varying concentrations showing (**a**) EL spectra, (**b**) current density–voltage, (**c**) luminance–voltage, (**d**) power efficacy–luminance (**e**) current efficacy–luminance and (**f**) luminance–current-density characteristics; Figure S2: The electroluminescent (EL) properties of the device with emitter RB-09 doped in CBP host matrix at varying concentrations showing (**a**) EL spectra, (**b**) current density–voltage, (**c**) luminance–voltage, (**d**) power efficacy–luminance (**e**) current efficacy–luminance and (**f**) luminance–current-density characteristics; Figure S3: The electroluminescent (EL) properties of the device with emitter RB-10 doped in CBP host matrix at varying concentrations showing (**a**) EL spectra, (**b**) current density–voltage, (**c**) luminance–voltage, (**d**) power efficacy–luminance (**e**) current efficacy–luminance and **(f)** luminance–current-density characteristics; Figure S4: The electroluminescent (EL) properties of the device with emitter RB-11 doped in CBP host matrix at varying concentrations showing (**a**) EL spectra, (**b**) current density–voltage, (**c**) luminance–voltage, (**d**) power efficacy–luminance (**e**) current efficacy–luminance and **(f)** luminance–current-density characteristics; Figure S5: TGA curve of the objective material RB-08; Figure S6: TGA curve of the objective material RB-09; Figure S7: TGA curve of the objective material RB-10;Table S1: The electroluminescent (EL) characteristics of the devices utilizing emitters RB-08, RB-09, RB-10, and RB-11 doped in the CBP host matrix. These characteristics included the turn-on voltage at a luminance greater than 1 cd m^−2^, power efficacy, current efficacy, external quantum efficiency, CIE coordinates, and maximum luminance. The devices were evaluated at different emitter concentrations; Reference [23] is cited in Supplementary Materials file.

## Sample Availability

Samples of the compounds RB-08, RB-09, RB-10, or RB-11 are available from the authors.
