# Supplementary material for: Naphtalimide-Based Bipolar Derivatives Enabling High-Efficiency OLEDs
Source: Molecules. 2023 Aug 12;28(16):6027. doi: 10.3390/molecules28166027 (PMC10458866; doi:10.3390/molecules28166027)
Supplement: Supplementary file 1 [file molecules-28-06027-s001.zip › molecules-2544794-supplementary.pdf]

Naphtalimide-Based Bipolar Derivatives Enabling High-Efficiency OLEDs

Raminta Beresneviciute [1], Prakalp Gautam [2], Mangey Ram Nagar [2], Gintare Krucaite [1], Daiva Tavgeniene [1],
Jwo-Huei Jou [2,*] and Saulius Grigalevicius [1,*]

[1] Department of Polymer Chemistry and Technology, Kaunas University of Technology, Radvilenu Plentas 19, LT50254 Kaunas, Lithuania
[2] Department of Materials Science and Engineering, National Tsing Hua University, No. 101, Section 2, Guangfu Rd., East District, Hsinchu 30013, Taiwan
* Correspondence: jjou@mx.nthu.edu.tw (J.-H.J.); saulius.grigalevicius@ktu.lt (S.G.)

Table of Contents

1. Starting materials and spectral data of the synthesized materials
2. Electroluminescent properties
3. TGA curves of the objective materials
4. References

Starting materials and spectral data of the synthesized materials

Carbazole (**1**), phenylboronic acid, naphthalene-1-boronic acid, 9H-carbazole-9-(4-phenyl) boronic acid pinacol ester, 4-(diphenylamino)phenylboronic acid, 4-bromo-1,8-naphtalic anhydride (**6**), 3-amino-9-ethylcarbazole, bis(triphenylphosphine)palladium(II) dichloride, 18-crown-6, KOH, KI, KIO_3, K_2CO_3, Cu, CuI, Na_2SO_4, chloroform, tetrahydrofuran and dimethylformamide were purchased from Aldrich and used as received.

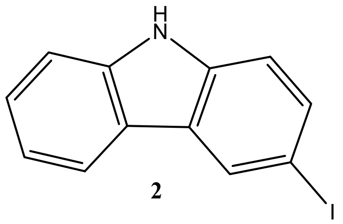

2

3-Iodo-9H-carbazole (**2**) was prepared from commercially obtained 9H-carbazole in Tucker iodination procedure [1].

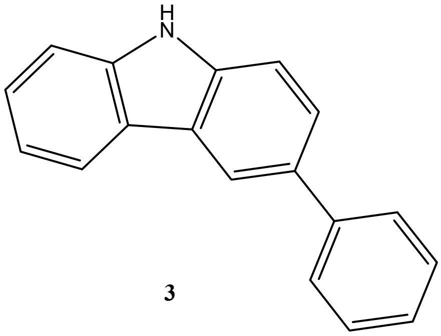

3

3-Phenyl-9H-carbazole (**3**). ^{1}H NMR (400 MHz, $CDCl_3$-d_6, δ, m.d.): 8.47 (s, 1H, Ar), 8.04 (d, 4H, J = 7.6Hz, Ar), 7.69 (d, 2H, J = 8.4Hz, Ar), 7.45 (s, 1H, Ar), 7.47-7.23 (m, 5H, Ar). ^{13}C NMR (100 MHz, $CDCl_3$-d_6, δ, m.d.): 139.48, 139.46, 138.59, 138.56, 134.12, 134.07, 129.29, 129.25, 126.60, 122.11, 120.49, 119.97, 112.63, 112.60, 110.81, 110.75. MS (APCI+, 20 V): 244.30 ([M+H], 100 %).

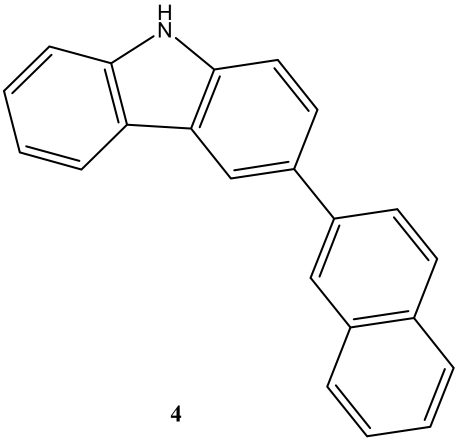

4

3-(1-Naphtyl)-9H-carbazole (**4**). ^{1}H NMR (400 MHz, $CDCl_3$-d_6, δ, m.d.): 8.25 (d, 1H J = 1.0 Hz, Ar), 8.12 (d, 2H, J = 7.7 Hz, Ar), 8.06 (d, 1H, J = 8.5 Hz, Ar), 7.99 (d, 1H, J = 8.1 Hz, Ar), 7.93 (dd, 1H, J_1 = 7.4 Hz, J_2 = 1.7 Hz, Ar), 7.65-7.44 (m, 9H, Ar). ^{13}C NMR (100 MHz, $CDCl_3$-d_6, δ, m.d.): 141.06, 139.96, 138.80, 133.93, 132.25, 132.15, 128.29, 128.20, 127.43, 127.29, 126.45, 126.09, 125.97, 125.74, 125.49, 123.44, 123.38, 121.79, 120.47, 119.66, 110.77, 110.29. MS (APCI+, 20 V): 294.12 ([M+H], 100 %).

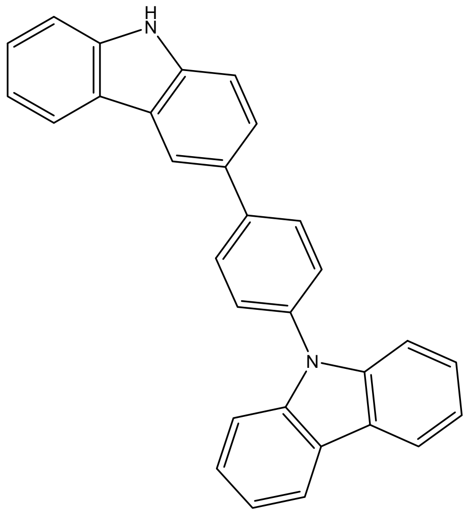

5

3-[4-(Carbazol-9-yl)phenyl]-9H-carbazole (**5**). ^{1}H NMR (400 MHz, $CDCl_3$-*d₆*, δ, m.d.): 8.43 (s, 1H, Ar), 8.21 (dd, 3H, J_1 = 7.7, J_2 = 3.7 Hz, Ar), 8.16 (s, 1H, Ar), 7.97 (d, 2H, J = 8.3 Hz, Ar), 7.80 (dd, 1H, J_1 = 8,4, J_2 = 1.6 Hz, Ar), 7.70 (d, 2H, J = 8.3 Hz, Ar), 7.60-7.52 (m, 2H, Ar), 7.50 (d, 1H, J = 3.3 Hz, Ar), 7.47 (d, 1H, J = 7.8 Hz, Ar), 7.38-7.32 (m, 3H, Ar), 7.29 (s, 1H, Ar). ^{13}C NMR (100 MHz, $CDCl_3$-d_6, δ, m.d.): 141.30, 141.00, 140.02, 139.15, 136.11, 132.08, 128.61, 127.41, 126.24, 126.02, 125.97, 125.37, 124.06, 123.40, 120.45, 120.33, 119.91, 119.77, 118.94, 111.01, 110.83, 109.92. MS (APCI+, 20 V): 409.17 ([M+H], 100 %).

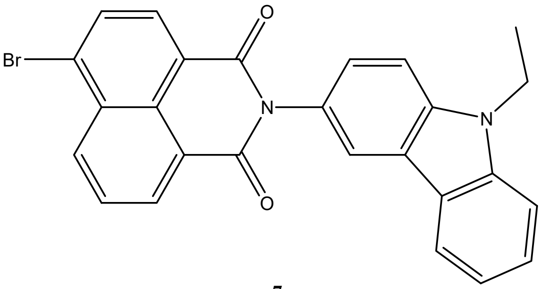

7

N-(9-Ethylcarbazol-3-yl)-4-bromo-1,8-naphtalymide (**7**). ^{1}H NMR (400 MHz, $CDCl_3$-d_6, δ, m. d.): 8,65 (d, 1H, J = 7,2 Hz, Ar), 8,55 (d, 1H, J = 8,4 Hz, Ar), 8,4 (d, 1H, J = 7,6 Hz, Ar), 8,01–7,93 (m, 3H, Ar), 7,81 (t, 1H, J = 8 Hz, Ar), 7,48 (d, 1H, J = 8,8 Hz, Ar), 7,42–7,29 (m, 3H, Ar), 7,18–7,11 (m, 1H, Ar), 4,33 (q, 2H, J = 7,2, $–CH_2–$), 1,4 (t, 3H, J = 7,2 Hz, $–CH_3$). ^{13}C NMR (100 MHz, $CDCl_3$-d_6, δ, m. d.): 164,48, 140,49, 139,74, 133,54, 132,46, 131,62, 131,23, 130,82, 130,58, 129,43, 128,21, 126,13, 126,06, 125,51, 123,68, 123,49, 122,81, 122,63,

120,79, 120,61, 119,08, 77,37, 77,05, 76,73, 37,77, 13,95. MS (APCI+, 20 V): 469,1 ([M+H]$^+$, 100 %).

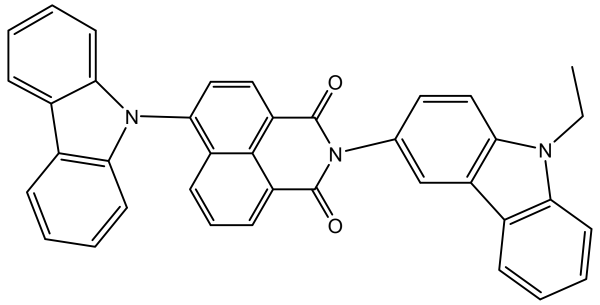

8

N-(9-Ethylcarbazol-3-yl)-4-(carbazol-9-yl)-1,8-naphtalimide (**8**). ^{1}H NMR (400 MHz, $CDCl_3$-*d6*, δ, m. d.): 8,79 (d, 1H, J = 7,6 Hz, Ar), 8,67 (d, 1H, J = 7,2 Hz, Ar), 8,17 (d, 1H, J = 7,2 Hz, Ar), 8,03 (s, 1H, Ar), 8,00 (d, 1H, J = 7,6 Hz, Ar), 7,85 (d, 1H, J = 7,6 Hz, Ar), 7,79 (d, 1H, J = 8 Hz, Ar), 7,59 (t, 1H, J = 7,6 Hz, Ar), 7,53 (d, 1H, J = 8,4 Hz, Ar), 7,44–7,23 (m, 7H, Ar), 7,17–7,14 (m, 2H, Ar), 7,01 (d, 2H, J = 7,6 Hz, Ar), 4,36 (q, 2H, J = 6,8 Hz, $–CH_2–$), 1,43 (t, 3H, J = 7,2 Hz, $–CH_3$). ^{13}C NMR (100 MHz, $CDCl_3$-*d6*, δ, m. d.): 164,81, 164,41, 141,79, 140,58, 140,53, 139,78, 132,45,132,01, 130,42, 130,17, 129,24, 127,76, 127,50, 127,47, 126,44, 126,26, 126,10, 125,59, 123,85, 123,78, 123,73, 123,13, 122,85, 120,83, 120,81, 120,74, 120,66, 119,10, 110,05, 109,25, 108,68, 108,63, 77,37, 77,26, 77,05, 76,73, 37,80, 13,97. MS (APCI+, 20 V): 556,81 ([M+H]$^+$, 100 %).

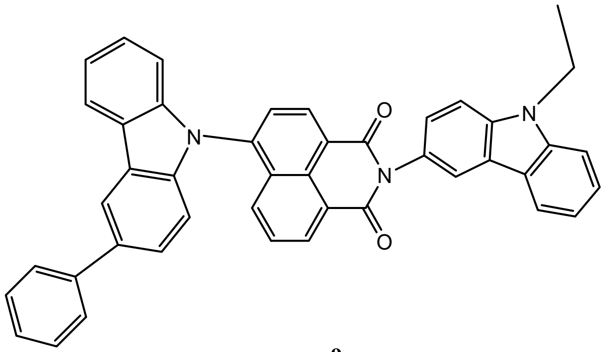

9

N-(9-Ethylcarbazol-3-yl)-4-(3-phenylcarbazol-9-yl)-1,8-naphtalimide (**9**). ^{1}H NMR (400 MHz, $CDCl_3$-*d6*, δ, m. d.): 8,83 (d, 1H, J = 7,6 Hz, Ar), 8,69 (d, 1H, J = 7,2 Hz, Ar), 8,62 (d, 1H, J = 7,2 Hz, Ar), 8,37 (s, 1H, Ar), 8,23-8,21 (m, 2H, Ar), 8,04–7,92 (m, 3H, Ar), 7,84 (d, 1H, J = 8,4 Hz, Ar), 7,74 (t, 1H, J = 8,00 Hz, Ar), 7,66 (t, 2H, J = 9,6 Hz, Ar), 7,61–7,49 (m, 3H, Ar), 7,45–7,31 (m, 8H, Ar), 4,38 (q, 2H, J = 7,6 Hz, $-CH_2-$), 1,44 (t, 3H, J = 8,00 Hz, -

CH_3).^{13}C NMR (100 MHz, $CDCl_3$-*d6*, δ, m. d.): 170,74, 169,17, 166,83, 166,14, 164,42, 158,32, 157,41, 155,83, 155,01, 154,63, 153,03, 151,31, 149,36, 147,85, 145,45, 144,62, 143,04, 142,19, 139,78, 139,05, 135,89, 130,82, 128,89, 128,65, 127,82, 125,64, 121,72, 119,10, 118,86, 117,13, 116,29, 114,50, 112,16, 110,21, 108,40, 77,34, 77,03, 76,71, 37,54, 13,96. MS (APCI+, 20 V): 632,19 ($[M+H]^+$, 100 %).

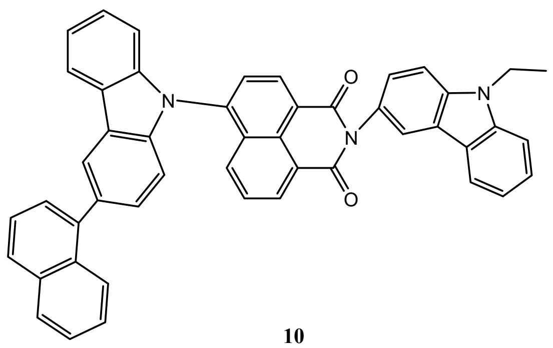

10

N-(9-Ethylcarbazol-3-yl)-4-[3-(1-naphthyl)carbazol-9-yl]-1,8-naphtalimide (**10**). ^{1}H NMR (400 MHz, $CDCl_3$-*d6*, δ, m. d.): 8,85 (d, 1H, J = 8,00 Hz, Ar), 8,71 (d, 1H, J = 6,8 Hz, Ar), 8,62 (d, 1H, J = 7,2 Hz, Ar), 8,28 (s, 1H, Ar), 8,22 (d, 1H, J = 8,4 Hz, Ar), 8,16 (d, 1H, J = 7,6 Hz, Ar), 8,04–7,83 (m, 6H, Ar), 7,74 (t, 1H, J = 7,6 Hz, Ar), 7,67 (t, 1H, J = 8,00 Hz, Ar), 7,55–7,29 (m, 9H, Ar), 7,17–7,12 (m, 2H, Ar), 7,05 (d, 1H, J = 8,00 Hz, Ar), 4,38 (q, 2H, J = 7,2 Hz, $-CH_2-$), 1,43 (t, 3H, J = 8,00 Hz, $-CH_3$). ^{13}C NMR (100 MHz, $CDCl_3$-*d6*, δ, m. d.): 164,41, 163,74, 163,05, 162,69, 162,00, 161,11, 160,88, 159,82, 157,87, 154,78, 154,63, 152,22, 151,77, 150,70, 149,36, 145,22, 144,16, 142,20, 139,79, 137,38, 135,88, 128,05, 126,08, 125,85, 125,63, 124,95, 124,36, 123,68, 121,27, 120,83, 119,32, 119,11, 118,84, 117,59, 113,21, 111,47, 110,79, 110,56, 110,21, 77,34, 77,03, 76,71, 31,60, 13,96. MS (APCI+, 20 V): 682,97 ($[M+H]^+$, 100 %).

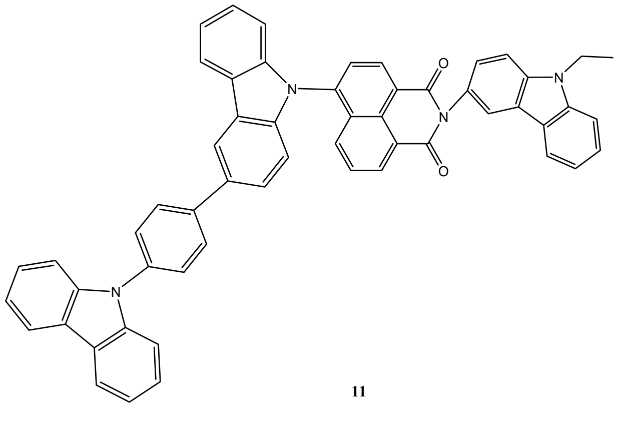

11

N-(9-Ethylcarbazol-3-yl)-4-{3-[4-(carbazol-9-yl)phenyl]carbazol-9-yl}-1,8-naphtalimide (**11**). ^{1}H NMR (400 MHz, $CDCl_3$-*d_6*, δ, m. d.): 8,82 (d, 1H, J = 7,6 Hz, Ar), 8,69 (d, 1H, J = 6,8 Hz, Ar), 8,60 (d, 1H, J = 6,8 Hz, Ar), 8,25–8,17 (m, 2H, Ar), 8,10 (d, 2H, J = 7,6 Hz, Ar), 8,00–7,79 (m, 5H, Ar), 7,73–7,60 (m, 4H, Ar), 7,53–7,30 (m, 10H, Ar), 7,25–7,04 (m, 5H, Ar), 4,34 (q, 2H, J = 6,8 Hz, $–CH_2–$), 1,60 (t, 3H, J = 7,2 Hz, $–CH_3$). ^{13}C NMR (100 MHz, $CDCl_3$-*d_6*, δ, m. d.): 165,07, 164,78, 164,36, 142,29, 141,42, 140,94, 140,81, 140,54, 140,49, 139,79, 139,71, 136,48, 134,19, 133,47, 132,52, 132,01, 131,79, 131,63, 130,33, 130,21, 129,21, 128,69, 128,61, 127,89, 127,48, 127,05, 126,83, 126,13, 126,00, 125,91, 125,65, 124,54, 123,91, 123,68, 123,46, 123,28, 123,09, 122,84, 121,10, 120,81, 120,68, 120,38, 119,21, 119,13, 119,03, 110,51, 110,29, 109,89, 108,70, 108,62, 77,38, 77,26, 77,06, 76,74, 37,75, 13,95. MS (APCI+, 20 V): 797,58 ($[M+H]^+$, 100 %).

Electroluminescent properties

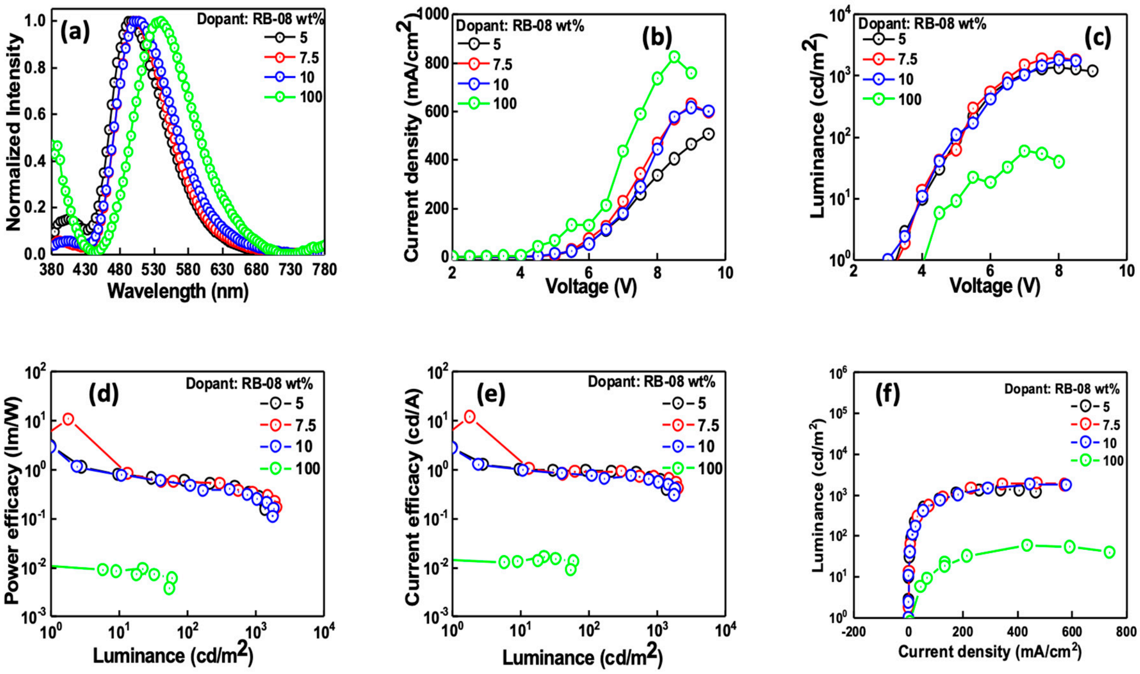

Figure S1. The electroluminescent (EL) properties of the device with emitter RB-08 doped in CBP host matrix at varying concentrations showing (**a**) EL spectra, (**b**) current density–voltage, (**c**) luminance–voltage, (**d**) power efficacy–luminance (**e**) current efficacy–luminance and **(f)** luminance–current-density characteristics.

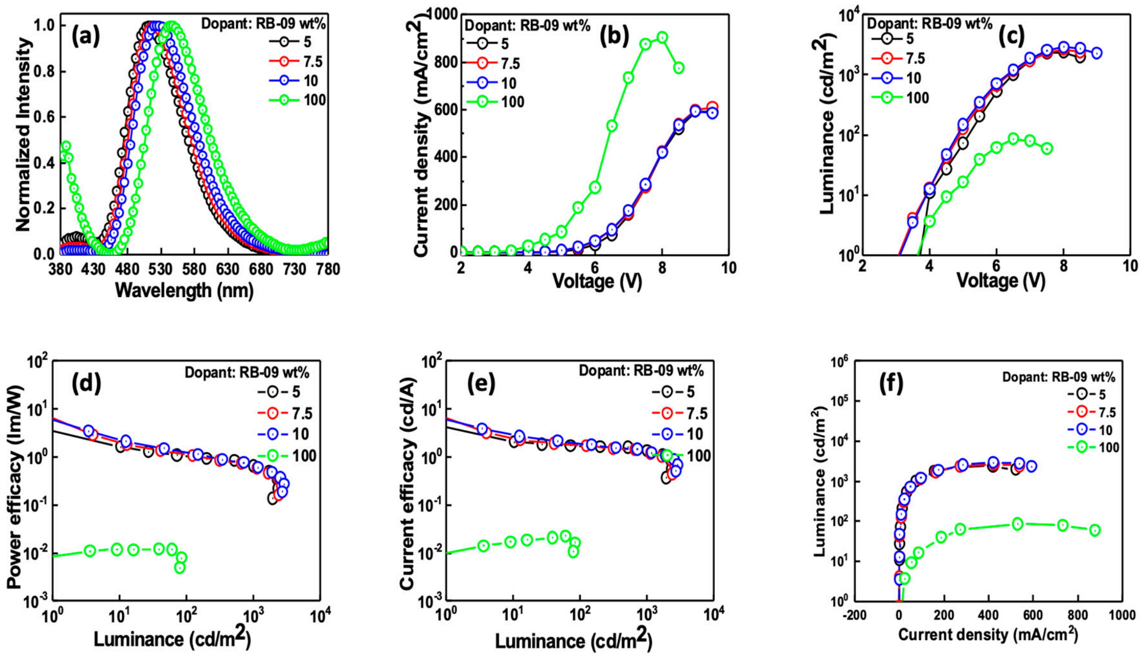

Figure S2. The electroluminescent (EL) properties of the device with emitter RB-09 doped in CBP host matrix at varying concentrations showing (**a**) EL spectra, (**b**) current density–voltage, (**c**) luminance–voltage, (**d**) power efficacy–luminance (**e**) current efficacy–luminance and **(f)** luminance–current-density characteristics.

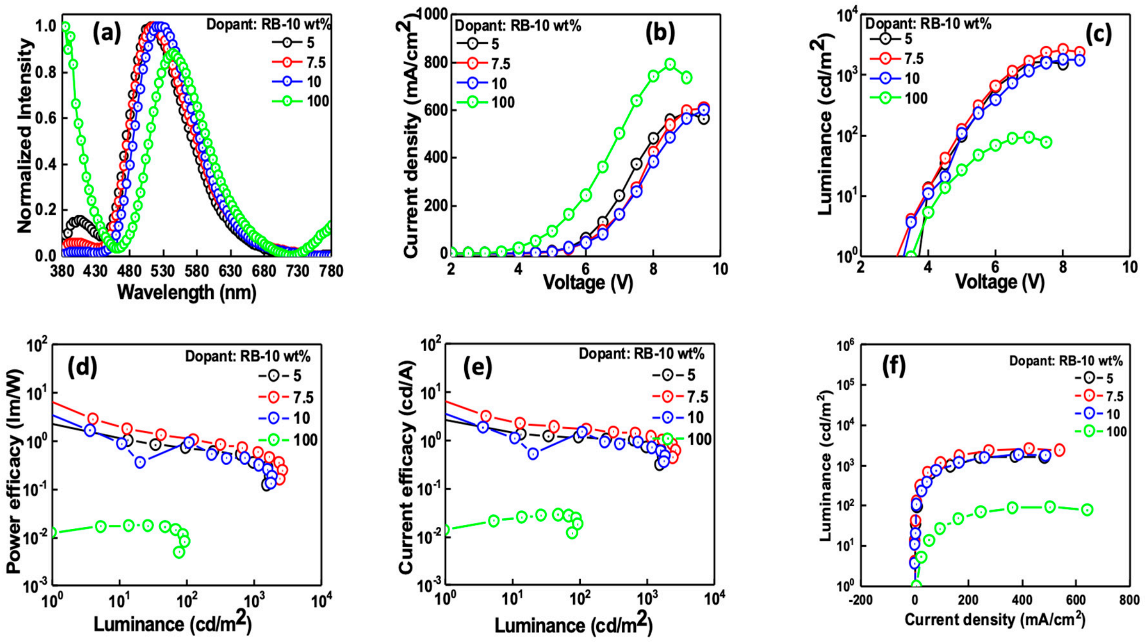

Figure S3. The electroluminescent (EL) properties of the device with emitter RB-10 doped in CBP host matrix at varying concentrations showing (**a**) EL spectra, (**b**) current density–voltage,

(**c**) luminance–voltage, (**d**) power efficacy–luminance (**e**) current efficacy–luminance and **(f)** luminance–current-density characteristics.

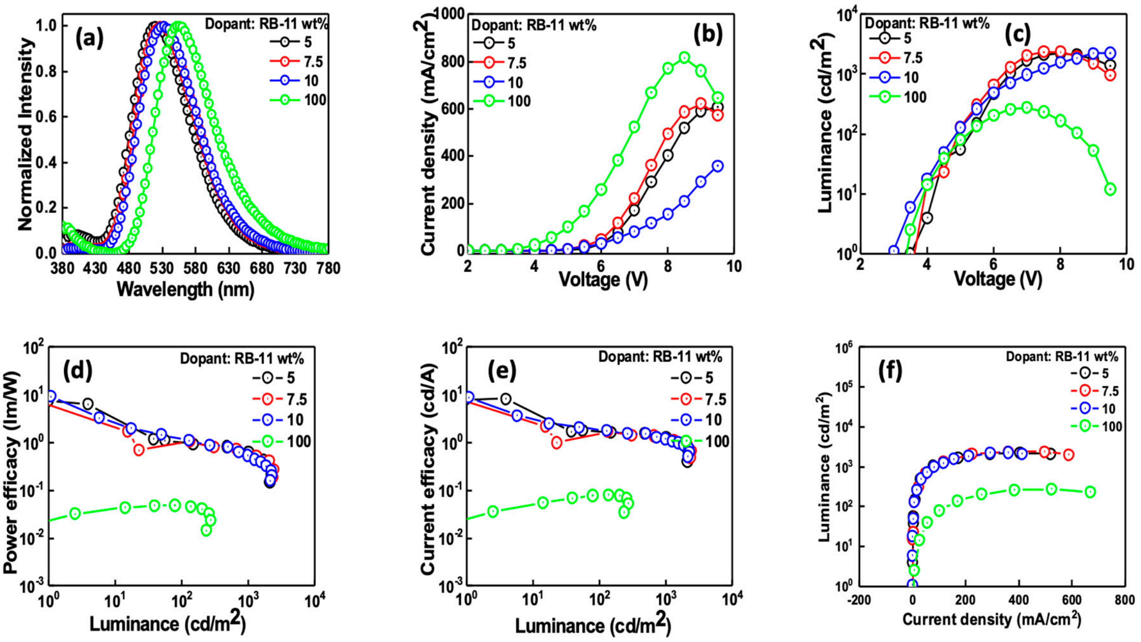

Figure S4. The electroluminescent (EL) properties of the device with emitter RB-11 doped in CBP host matrix at varying concentrations showing (**a**) EL spectra, (**b**) current density–voltage, (**c**) luminance–voltage, (**d**) power efficacy–luminance (**e**) current efficacy–luminance and **(f)** luminance–current-density characteristics.

Table S1. The electroluminescent (EL) characteristics of the devices utilizing emitters RB-08, RB-09, RB-10, and RB-11 doped in the CBP host matrix. These characteristics included the turn-on voltage at a luminance greater than 1 cd m^{-2}, power efficacy, current efficacy, external quantum efficiency, CIE coordinates, and maximum luminance. The devices were evaluated at different emitter concentrations

**Emitter**	**Dopants Concentration (wt%)**	**Driving Voltage (V)**	**Operation Voltage (V)**	**Power efficiency (lm/W)**	**Current efficiency (cd/A)**	**EQE (%)**	**CIE**	**Max Luminance (cd/m²)**
			**@100, 1000 cd/m² and maximum**					
**RB-08**	**5**	4.0	5.0/ 6.8/ 3.0	0.6/ 0.3/ 3.0	1.0/ 0.7/ 2.9	0.4/ 0.3/ 1.1	(0.22, 0.42)/ (0.21, 0.36)/ -	1359
	**7.5**	3.9	5.1/ 6.6/ 3.5	0.6/ 0.3/ 10.9	0.9/ 0.7/ 12.2	0.4/ 0.3/ 1.0	(0.23, 0.47)/ (0.21, 0.43)/ -	2007
	**10**	4.0	4.9/ 6.9/ 3.0	0.5/ 0.3/ 2.9	0.8/ 0.6/ 2.8	0.33/ 0.2/ 1.1	(0.25, 0.47)/ (0.22, 0.42)/ -	1823
	**100**	5.0	- / - / -	- / - / -	- / - / -	- / - / -	- / - / -	59

**RB-09**	**5**	4.0	5.1/ 6.5/ 3.5	1.1/ 0.7/ 6.2	1.7/ 1.4/ 6.9	0.7/ 0.5/ 1.6	(0.27, 0.5)/ (0.24, 0.45)/ -	2364
	**7.5**	3.8	4.8/ 6.3/ 3.0	1.2/ 0.6/ 7.5	1.8/ 1.3/ 7.2	0.8/ 0.5/ 2.1	(0.29, 0.53)/ (0.26, 0.49)/ -	2655
	**10**	3.9	4.8/ 6.3/ 3.1	1.3/ 0.7/ 6.8	2/ 1.3/ 6.5	0.6/ 0.5/ 2.8	(0.31, 0.55)/ (0.28, 0.51)/ -	2879
	**100**	4.3	- / - / -	- / - / -	- / - / -	- / - / -	- / - / -	84
**RB-10**	**5**	3.9	5/ 6.5/ 3.5	0.7/ 0.4/ 4.2	1.2/ 0.7/ 4.6	0.6/ 0.4/ 1.6	(0.26, 0.48)/ (0.23, 0.41)/ -	1687
	**7.5**	3.8	4.8/ 6.3/ 3.0	1.2/ 0.6/ 7.5	1.8/ 1.3/ 7.2	0.5/ 0.3/ 0.8	(0.28, 0.51)/ (0.24, 0.45)/ -	2655

	**10**	3.9	5.0/ 6.8/ 3.1	0.9/ 0.4/ 6.9	1.4/ 0.8/ 6.7	0.4/ 0.3/ 0.9	(0.31, 0.55)/ (0.28, 0.51)/ -	1827
	**100**	4.3	- / - / -	- / - / -	- / - / -	- / - / -	- / - / -	93
**RB-11**	**5**	4.1	5.2/ 6.5/ 3.2	1.0/ 0.6/ 7.7	1.7/ 1.3/ 7.9	0.6/ 0.5/ 3.3	(0.29, 0.52)/ (0.26, 0.46)/ -	2212
	**7.5**	3.8	4.9/ 6.3/ 3.5	1/ 0.6/ 9	1.5/ 1.2/ 10	0.7/ 0.5/ 2.4	(0.31, 0.53)/ (0.28, 0.49)/ -	2377
	**10**	3.7	4.8/ 7.0/ 3.0	1.2/ 0.5/ 9.3	1.9/ 1.2/ 8.8	0.6/ 0.4/ 2.9	(0.33, 0.55)/ (0.29, 0.51)/ -	2253
	**100**	4.3	5.2 / - / -	-/ - / -	- / - / -	- / - / -	(0.39, 0.51) / - / -	477

TGA curves of the objective materials

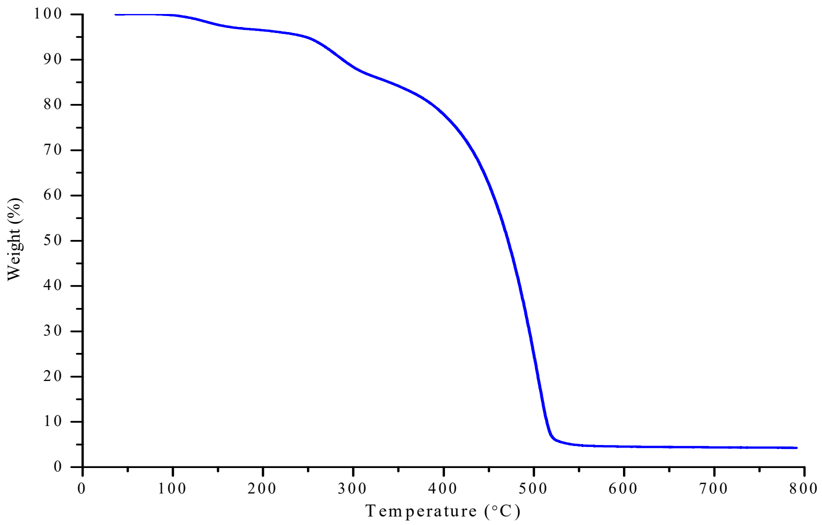

Figure S5. TGA curve of the objective material RB-08.

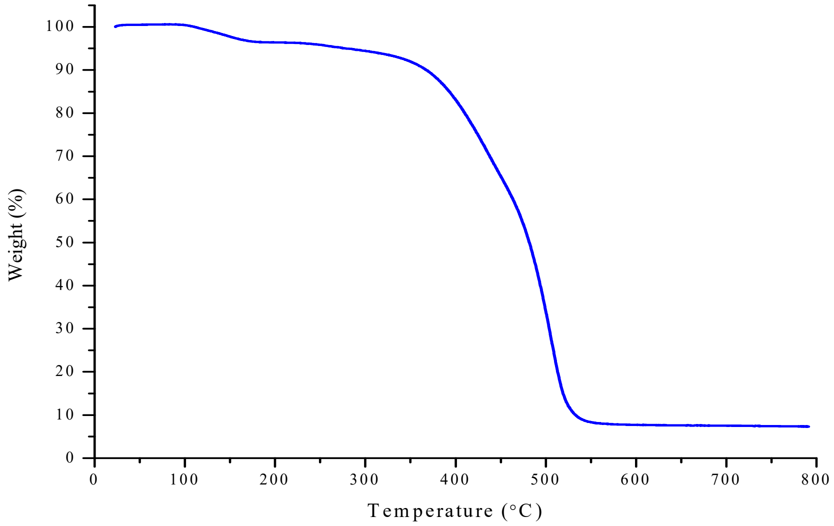

Figure S6. TGA curve of the objective material RB-09.

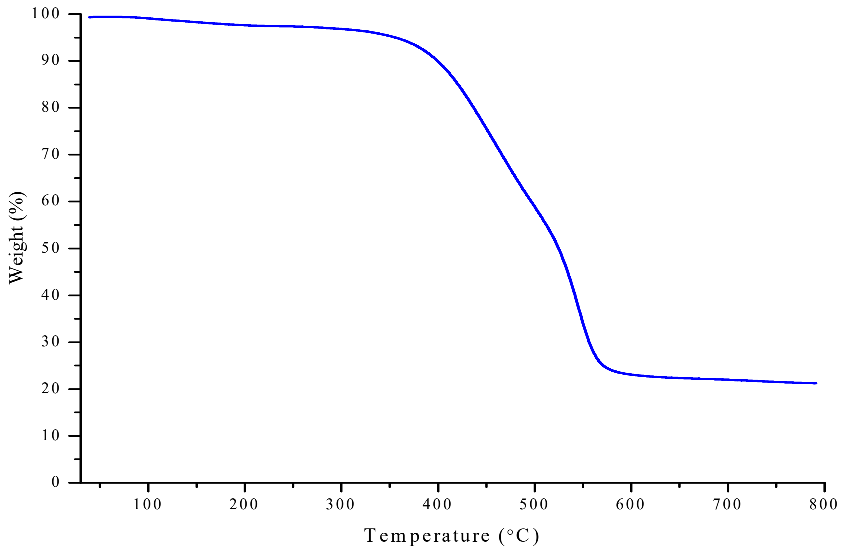

Figure S7. TGA curve of the objective material RB-10.

References:

1. Tucker, S. H. (1926). LXXIV.—Iodination in the carbazole series. *Journal of the Chemical Society*, *129*, 546-553.
